# A Genome-Wide Association Study of Highly Heritable Agronomic Traits in Durum Wheat

**DOI:** 10.3389/fpls.2019.00919

**Published:** 2019-07-17

**Authors:** Shubin Wang, Steven Xu, Shiaoman Chao, Qun Sun, Shuwei Liu, Guangmin Xia

**Affiliations:** ^1^Key Laboratory of Plant Development and Environmental Adaptation Biology, Ministry of Education, School of Life Sciences, Shandong University, Qingdao, China; ^2^United States Department of Agriculture-Agricultural Research Service (USDA-ARS), Cereal Crops Research Unit, Edward T. Schafer Agricultural Research Center, Fargo, ND, United States; ^3^Department of Plant Sciences, North Dakota State University, Fargo, ND, United States

**Keywords:** wheat, durum wheat, agronomic traits, drought tolerance, genome-wide association study, evolutionary divergence

## Abstract

Uncovering the genetic basis of key agronomic traits, and particularly of drought tolerance, addresses an important priority for durum wheat improvement. Here, a genome-wide association study (GWAS) in 493 durum wheat accessions representing a worldwide collection was employed to address the genetic basis of 17 agronomically important traits and a drought wilting score. Using a linear mixed model with 4 inferred subpopulations and a kinship matrix, we identified 90 marker-trait-associations (MTAs) defined by 78 markers. These markers could be merged into 44 genomic loci by linkage disequilibrium (*r*^2^ > 0.2). Based on sequence alignment of the markers to the reference genome of bread wheat, we identified 14 putative candidate genes involved in enzymes, hormone-response, and transcription factors. The GWAS in durum wheat and a previous quantitative trait locus (QTL) analysis in bread wheat identified a consensus QTL locus.4B.1 conferring drought tolerance, which was further scanned for the presence of potential candidate genes. A haplotype analysis of this region revealed that two minor haplotypes were associated with both drought tolerance and reduced plant stature, thought to be the effect of linkage with the semi-dwarfing gene *Rht-B1*. Haplotype variants in the key chromosome 4B region were informative regarding evolutionary divergence among durum, emmer and bread wheat. Over all, the data are relevant in the context of durum wheat improvement and the isolation of genes underlying variation in some important quantitative traits.

## Introduction

The bulk of the wheat cropped across the world is represented by either hexaploid bread wheat (*Triticum aestivum* ssp. *aestivum*) or tetraploid durum wheat (*Triticum turgidum* ssp. *durum*). The latter shares two of the former’s three sub-genomes, both of which evolved from wild emmer wheat (*T. turgidum* ssp. *dicoccoides*) by way of cultivated emmer wheat (*T. turgidum* ssp. *dicoccum*; reviewed by Faris, 2014). However, durum and bread wheat are distinct in aspects of diverse phenotypic features, due to the genetic divergence resulting from their independent domestication and difference in ploidy level. The production of durum wheat is dwarfed by that of bread wheat, but remains nevertheless of considerable economic importance, as its grain is better suited than that of bread wheat for the manufacture of pasta, couscous and other semolina products.

Substantial research efforts have been devoted to determining the genetic basis of yield-related traits in bread wheat, such as plant height, heading date, morphological aspects with flag leaf, panicle and grain (e.g.; Quarrie et al., 2006; Kuchel et al., 2007; Kumar et al., 2007; Gegas et al., 2010; Wang et al., 2011; Wu et al., 2012; Würschum et al., 2017, 2018). However, the extent of this effort in durum wheat has been much more limited (e.g., Maccaferri et al., 2008; Peleg et al., 2009; Golabadi et al., 2011; Roncallo et al., 2017). The USDA-ARS National Small Grain Collection (NSGC, Aberdeen, ID, United States) conserves over 8,000 entries and represents the global diversity of durum wheat (Chao et al., 2017). A core subset has been assembled from this collection, comprising 493 entries with spring growth habit, of which 235 are classed as landraces, 77 as breeding lines, 55 as released cultivars, leaving 126 of unknown breeding status (Aoun et al., 2016; Chao et al., 2017). The size of this panel is appropriate for conducting genome-wide association studies (GWAS), a method which has been applied with some success to reveal the genetic basis of some key agronomic traits in bread wheat (e.g., Liu Y. et al., 2017; Sun et al., 2017; Würschum et al., 2017, 2018). The application of GWAS in durum wheat has to date been more limited, with most of these studies focused on geographically specific diversity and associated with limited sample size (e.g., Maccaferri et al., 2010; Mengistu et al., 2016; Kidane et al., 2017; Soriano et al., 2017; Mangini et al., 2018; Sukumaran et al., 2018).

The durum crop is typically rain-fed and frequently exposed to moisture deficiency in most of the durum-growing areas (Habash et al., 2009). For example, about 65% of the durum growing areas in the Mediterranean, which account for about 40% of global durum cultivated areas, is distributed in arid and semi-arid land with low rainfall below 350 mm (Nachit, 1994; Bassi and Sanchez-Garcia, 2017). Since the early 1990s, durum production in the United States has gradually moved from humid areas in the east (e.g., eastern North Dakota, United States) to the dry areas in the west (e.g., western North Dakota and eastern Montana, United States) due to severe outbreaks of Fusarium head blight (North Dakota Wheat Commission^1^). Drought stress can adversely affect grain yield of the wheat crop at various growth stages from germination to grain filling (Kizilgeçi et al., 2017). Drought at wheat early development stage can especially inhibit seedling vigor, tillering and root development, thus reducing biomass accumulation and grain yield (Kizilgeçi et al., 2017; Zhao et al., 2019). Compared with bread wheat with winter-type varieties dominating globally, modern durum varieties around the world are mostly spring type or semi-winter type (Matsuo, 1994), typically with 3–4 months of growth periods. In such a short life cycle, drought stress at the seedling stage probably reduces grain yield more seriously.

As drought stress is the major limiting factor for global durum production (Grant et al., 2012), any enhancement in drought tolerance of durum wheat would represent a significant contribution to food security and farmer’s income. Breeding for drought tolerance (DT) is conventionally achieved by selection for yield in a target environment (Sukumaran et al., 2018), but success is hampered by the poor heritability of yield, difficulties in assuring homogeneity in the environment and the importance of genotype by environment interactions (Habash et al., 2009). A potentially attractive alternative strategy for DT selection is based on indirect traits, such as water use efficiency, leaf water content, leaf senescence, and root architecture, for which variation can be correlated with grain yield. Several such traits have been identified (Gupta et al., 2017).

Here, a durum panel with 493 entries was phenotyped with respect to a number of important morphological characters and a drought-related trait, and the resulting data merged with an extensive SNP (single nucleotide polymorphism)-based genotypic data set to conduct a GWAS, focusing on DT. The analysis has provided valuable information to understand the major genetic components of important agricultural traits and a more detailed haplotype and candidate gene of DT in durum.

## Materials and Methods

### Plant Materials

The line information and genotype data of the 493 durum wheat entries were reserved in the T3/Wheat database^2^. This panel has previously been exploited to identify the genetic basis of resistance against certain foliar pathogens (Aoun et al., 2016; Chao et al., 2017). In addition, genotypic data from a domesticated emmer wheat (Sun, 2015) and a bread wheat (Cavanagh et al., 2013) diversity panel was used to infer the genetic divergence of identified quantitative trait loci (QTL). All three diversity panels were genotyped using the wheat 9k iSelect assay (Cavanagh et al., 2013). Two F_2_ populations were developed to verify partial trait-associated loci or markers detected by GWAS. For each population, the parental lines (PI520392/PI210912 and PI191571/CITR14814) were selected from the durum diversity panel and, for each population, 120 F_2_ plants coupled with their F_3_ progenies were sampled for linkage analysis.

### Evaluation of Agronomic Traits

The durum wheat panel was evaluated for the following 18 morphological traits: plant height (PH), heading date (HD), plant waxiness (WX), glume pubescence (GP), glume color (GC), flag leaf length (FLL), flag leaf width (FLW), flag leaf length/width ratio (FLR), flag leaf angle (FLA), panicle length (PL), spikelet number per spike (SN), panicle compactness (PC), single kernel weight (KW), grain length (GL), grain width (GW), grain length/width ratio (GR), grain projection area (GA) and factor form density (FFD).

Of the 18 traits, 13 (the exceptions were WX, FLL, FLW, FLR, and FLA) were measured in 2012 and 2013 at North Dakota State University (NDSU, Fargo, ND, United States) research site at Prosper (46.9630°N, 97.0198°W; soil type: Perella-Bearden silty clay loam complex), and in 2014 at Shandong University research site (SDU, Jinan Shandong, China; 36.6489°N, 117.0290°E; soil type: brown loam). Fielded trails at the NDSU research site in 2012 (planting date: April 12) and 2013 (planting date: May 08) applied an unduplicated augmented design with four checks in each block (single-row plots, 3.2 m long with rows 30 cm apart) and no irrigation during the crop growing season. Average annual rainfall and snowfall in Fargo are documented as 573.53 and 914.40 mm, respectively (U.S. Climate Data^3^). The SDU field experiment (planting date: March 02) applied a randomized block design with four replicates (single-row plots, 2.0 m long with rows 30 cm apart) with no irrigation during the crop growing season. The annual average rainfall in Jinan was 521.20 mm. For the two F_2_ populations, their F_3_ families were planted at 2015 and 2016 growth season at Jinan using a randomized block design with three replicates.

The other five traits were evaluated only in the Jinan experiment. GP, GC, WX, and FLA were scored on a 1–9 scale. The traits’ value, except that involving grain morphology, represented the mean performance of five plants per replicate or trial. PC was calculated as the ratio of FN/PL. The grain morphology related traits were measured based on 30 uniform seeds. For the evaluation of GL, GW, and GA, 30 grains of each entry per replicate or trial were imaged and the resulting digital images were analyzed using software “ImageJ^4^.” FFD was calculated from the expression KW/GL^*^ GW, by following Gegas et al. (2010).

### Assessment of Drought Tolerance

For each entry, 15 uniform seedlings were planted in a soil-filled plastic cone, 6.4 cm in diameter at the open end and 25.4 cm in depth (total volume: 656 mL). Water was withheld once the plants had reached the three-leaf stage. The plants were re-watered until approximately 2/3 entries were wilted. Two days after re-watering, a drought wilting score was assigned using a 1–6 scale, where score 1 represented a completely wilted plant, 2 a plant in which the first three leaves were wilted, 3 a plant in which only the first two leaves were fully wilted and the third leaf was partially wilted, 4 a plant in which the first two leaves were fully wilted and the third leaf was not wilted, 5 a plant in which the first leaf was fully wilted, the second partially wilted and the third not wilted, and 6 a plant in which only the first leaf was wilted.

### Statistical and Bioinformatic Analyses

Summary statistics for each trait value was inferred in R (Ihaka and Gentleman, 1996). All trait values were fitted with a linear mixed model [trait ∼ line + 1|year + 1|location + 1|(block %in% location:year)] in R package Lme4^5^. The broad sense heritability was calculated as the ratio of total genetic variance to total phenotypic variance, and the best linear estimators were used for GWAS. The consensus linkage map of tetraploid wheat (Maccaferri et al., 2015) and the IWGSC RefSeq v1.0 genomic assembly (International Wheat Genome Sequencing Consortium, 2018^6^) were applied to assign a genomic location to each SNP marker. Pairwise linkage disequilibrium (LD, *r*^2^) was calculated using the package “genetics” in R (see text footnote 5). The program “Structure^7^” was used to assign ancestry proportion for each durum wheat entry based on a set of 366 unlinked markers (pairwise LD < 0.1, MAF > 0.05). The admixture model was fitted by varying the *K* parameter from 2 to 10. Marker-trait associations (MTAs) between each of the 18 morphological traits and DT and the set of informative SNPs were detected by fitting the mixed linear model (MLM) implemented in TASSEL software (Bradbury et al., 2007), treating the first three principal components (PCs) and HD as co-variates and using family relatedness as a random effect. A Bonferroni approach based on 168 haplotype blocks calculated by software Haploview^8^ was used to adjust the initial *p*, which resulting a –log10(*p*) = 3.53 equal to a false discovery rate (FDR) = 0.05. At the same time, a two-step approaches combining single- and multiple-locus LMM model was used to claim significant MTA (Wang et al., 2016). All MTAs satisfying either criterion were included in the final result. The haplotype network was inferred using TCS software (Clement et al., 2000). For linkage analysis in the F_2_ populations, dCAPS markers were developed based on the flanking sequences of the corresponding SNP sites (Supplementary Table S1). QTL analysis was carried out in R package R/qtl^9^.

## Results

### High Genetic Polymorphism and Long LD Decay in Global Durum Collection

**FIGURE 1:**
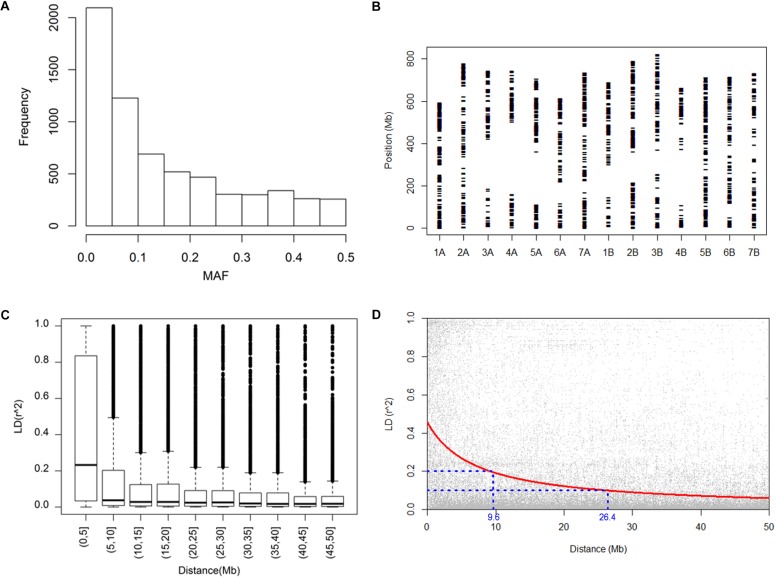
Genotypic characterization and linkage disequilibrium. **(A)** The distribution of the 6,538 SNP loci according to their minor allele frequency (MAF), **(B)** the chromosomal distribution of the SNP markers across the durum wheat genome, **(C)** a box plot representation of linkage disequilibrium, represented by the parameter r2, **(D)** the decay in linkage disequilibrium.

To examine the genetic polymorphism of the durum panel, we assessed the allelic variation of 8,136 SNP markers fixed in the wheat 9k iSelect assay (Wang et al., 2014). The SNP genotyping revealed signals at 6,538 SNP loci. A considerable accumulation was observed toward low polymorphic markers, in particular, about 32% of the detected markers had a minor allelic frequency (MAF) of less than 0.05 (Figure 1A). This was likely because of ascertainment bias, arising from the preponderance of bread wheats (as opposed to durum wheats) in the panel used to detect informative SNPs (Cavanagh et al., 2013). Overall, a set of 4,369 informative markers (MAF > 0.05 and missing value < 30%) were selected for this study. According to marker position, the polymorphic markers covers 90% of the genomic region, with a mean inter-marker distance of 2.6 Mb (Figure 1B). In this map, the only significant gaps present were in the vicinity of some of the centromeres. An analysis of pairwise LD showed that 42% of the marker pairs were associated with a *p*-value below 0.001, of which 38% recorded an *r*^2^ value of less than 0.1. Particularly high levels of LD were observed between markers separated by less than 5 Mb (Figure 1C). Based on the suggestion of Hill and Weir (1988), the decay in LD to 0.2 occurred within 9.6 Mb (Figure 1D), which is relatively smaller compared with previously reported LD (e.g., Bassi et al., 2019) in durum, mostly due to the large size of this panel. The high coverage of SNP polymorphism across the durum genome indicated an appropriate robustness but reduced resolution for GWAS.

### High Relatedness of Durum Wheat Accessions Independent From Breeding Status and Geographic Distribution

**FIGURE 2:**
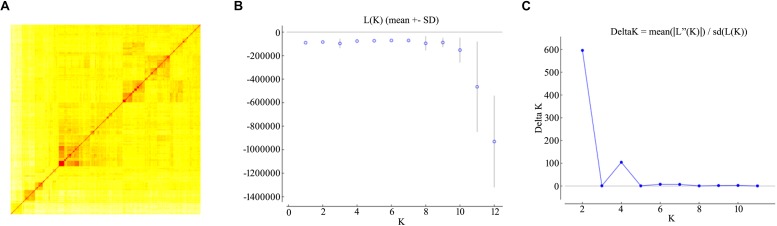
Population stratification of the durum diversity panel. **(A)** A kinship analysis inferred using a simple matching coefficient. In the heatmap, entries have been clustered using the Wad method. **(B,C)** STRUCTURE analysis of the durum wheat diversity panel. Log likelihood [L(K)] and ΔK values for each presumed ancestral population number (K) were derived using the admixture model; K was varied from 1 to 12 with five replications for each value of K. L(K) plateaux arose at *K* = 6 **(A)**, while ΔK showed a distinct and gave an inference of an optimal K at 2 **(B)**.

Population structure might bring false positive or negative marker-trait associations (MTAs) which should be considered as covariates in GWAS analysis. The population structure of the durum wheat panel based on the full set of informative SNPs has been previously described (Aoun et al., 2016; Chao et al., 2017). Due to the possible impact of linkage on the inference of genetic relationships, the genetic stratification issue was revisited by running an analysis using a sub-set of unlinked markers (pairwise LD < 0.2). A high pairwise relatedness among entries was established, with ∼80% of the comparisons producing an identity-by-state proportion of 0.6–0.8 (Figure 2A). The inferred genetic admixture and cluster assignment of the 493 entries suggested a structure comprised of two ancestral populations (Figures 2B,C) in agreement with the earlier analysis. A smaller peak in ΔK was also observed at *K* = 4. At both *K* = 2 and 4, the panel was highly heterogeneous with respect to both breeding status and geographical provenance, and harbored a large proportion of admixed entries: in the two sub-populations, respectively, 29 and 47% of the entries were associated lines with a *Q*-value of <0.7 (Supplementary Table S2).

### Phenotypic Variation for Agronomic Traits and DT

For assessment of drought tolerance, we first compared the drought wilting score with other seedling drought syndromes and DT index of yield components (Supplementary Figure S1). The drought wilting score is highly correlated with seedling drought syndromes (*r*^2^ = 0.73 with leaf water content, 0.36 with chlorophyll content, 0.46 with rolling index, and 0.64 with survival rate), and moderately correlated with some of the yield-related DT index (*r*^2^ = 0.26 with thousand seed weight, 0.05 with tiller number, and 0.07 with seed number per panicle). Therefore, this value could be a reflection to durum DT especially for early developmental stage.

**FIGURE 3:**
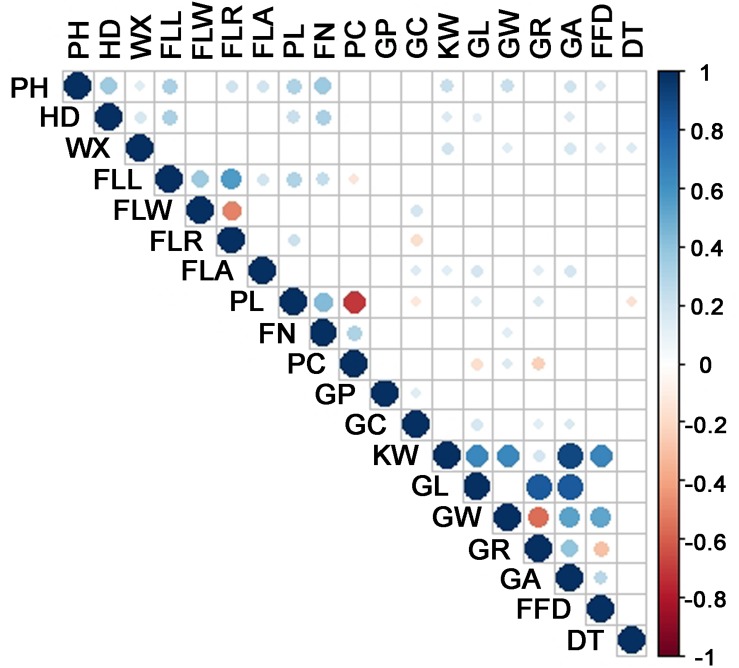
Inter-trait correlations, calculated using Pearson’s r coefficient. PH, plant height; HD, heading date; FLL, flag leaf length; FLW, flag leaf width; FLR, flag leaf length/width ratio; FLA, flag leaf angle; PL, spike length; SN, spikelet number per spike; PC, spike compactness; KW, single kernel weight; GL, grain length; GW, grain weight; GR, grain leaf length/width ratio; GA, grain projection area; FFD, factor form density; WX, waxiness; GP, glume pubescence; GC, glume color; DT, drought tolerance.

The durum wheat panel displayed a broad range of phenotype with respect to each of the traits (Table 1 and Supplementary Figure S2). The broad-sense heritability varied from 45 to 92 for the 17 quantitative traits – the exceptions were GP and GC, which were qualitative rather than quantitative in nature. A summary of the inter-trait correlations is given in Figure 3. The highest correlations involved pairs of traits within a single category, namely flag leaf, spike or grain morphology, but a few, mostly in the low to intermediate range, were recorded between unrelated traits. In particular, both PH and HD were correlated with a considerable number of other traits, while DT was positively correlated with both WX and PC, and negatively with both PH and PL.

**TABLE 1:** Variation displayed by the entries of the durum wheat panel with respect to 18 morphological traits and DT (a measure of drought tolerance).

**Trait**	**Measurement**	**Mean**	**Min**	**Max**	**SD**	**CV(%)**	***H*^2^**
PH	cm	98.32	40.00	150.00	21.58	21.95	0.87
HD	day	181.46	155.00	192.50	5.45	3.00	0.79
FLL	cm	25.75	14.83	62.08	4.55	17.67	0.69
FLW	cm	1.58	0.87	3.87	0.27	16.83	0.55
FLR	ratio	17.20	6.85	33.86	3.11	18.07	0.60
FLA	rank1–9	4.64	1.00	9.00	1.64	35.35	0.56
PL	cm	8.22	4.90	13.30	1.42	17.33	0.65
SN	count	18.77	10.20	45.00	2.43	12.94	0.73
PC	ratio	2.33	1.15	5.49	0.40	17.03	0.61
KW	mg	52.10	30.94	79.98	6.49	12.45	0.66
GL	mm	7.64	6.07	10.49	0.63	8.26	0.82
GW	mm	3.26	2.62	3.78	0.17	5.32	0.75
GR	ratio	2.35	1.68	3.32	0.24	10.03	0.69
GA	mm^2^	19.54	14.35	27.19	1.90	9.72	0.72
FFD	ratio	2.09	1.40	2.72	0.12	5.63	0.69
WX	rank1–9	6.53	1.00	9.00	1.85	28.37	0.92
GP	rank1–9	2.96	1.00	9.00	3.43	115.59	1.00
GC	rank1–9	3.32	1.00	9.00	3.56	107.24	0.98
DT	rank1–9	3.02	1.00	6.00	1.17	38.87	0.45

*SD, standard deviation; CV, coefficient of variation; H2, broad sense heritability; PH, plant height; HD, heading date; FLL, flag leaf length; FLW, flag leaf width; FLR, flag leaf length/width ratio; FLA, flag leaf angle; PL, spike length; SN, spikelet number per spike; PC, spike compactness; KW, single kernel weight; GL, grain length; GW, grain weight; GR, grain leaf length/width ratio; GA, grain projection area; FFD, factor form density; WX, waxiness; GP, glume pubescence; GC, glume color; DT, drought tolerance.*

With respect to seven of the agronomic traits, the elite breeding lines and/or cultivars differed as a group from the landraces (Table 2), reflecting the effect of intensive selection pressure. The elite materials scored lower for PH, HD, FLL, and FLA, and higher for PL and SN; however, there was no such differentiation with respect to any of the grain morphology traits. The DT score averaged over the set of breeding lines exceeded that of landraces (3.59 versus 2.95). However, as a group, the mean performance of PH and DT in the cultivars was inferior to that of the landraces.

**TABLE 2:** Analysis of variance with respect to 18 morphological traits and DT among the entries of the durum wheat panel according to their breeding status.

**Traits**	**Breading status**								***P*-value**	
	**Landrace**		**Breeding line**		**Cultivar**		**Unkown**			
PH	99.42	a	84.10	**b**	101.04	a	103.88	a	0.00	^∗∗∗^
HD	181.21	a	180.51	**b**	180.85	a	182.78	**c**	0.01	^*^
WX	6.47	a	6.85	a	6.71	a	6.39	a	0.28	
FLL	25.68	a	24.54	**b**	25.95	a	26.53	**c**	0.03	^*^
FLW	1.58	a	1.53	a	1.61	a	1.61	a	0.48	
FLR	17.12	a	16.70	a	17.64	a	17.38	a	0.60	
FLA	4.79	a	4.12	**b**	4.58	**c**	4.72	**c**	0.02	^*^
PL	8.07	a	8.13	**b**	8.19	**b**	8.57	**c**	0.02	^*^
SN	18.28	a	18.59	a	18.90	**b**	19.76	**c**	0.00	^∗∗∗^
PC	2.32	a	2.33	a	2.37	a	2.35	a	0.78	
GP	3.36	a	2.33	a	2.00	a	3.00	a	0.03	^*^
GC	3.84	a	2.82	a	2.33	a	3.00	a	0.02	^*^
KW	52.58	a	51.49	a	53.00	a	51.20	a	0.15	
GL	7.73	a	7.53	a	7.57	a	7.56	a	0.02	^*^
GW	3.25	a	3.27	a	3.29	a	3.25	a	0.35	
GR	2.39	a	2.31	a	2.30	a	2.33	a	0.02	^*^
GA	19.73	a	19.31	a	19.58	a	19.30	a	0.14	
FFD	2.09	a	2.09	a	2.12	a	2.08	a	0.22	
DT	2.95	a	3.59	**b**	2.68	a	2.90	a	0.00	^∗∗∗^

Trait abbreviations as given in Table 1. Means differing significantly from one another (^*^, ^∗∗∗^p < 0.01, 0.001) have been assigned a different lower case letter.

### The Genetic Basis of Agronomic Traits and DT Revealed by GWAS

**FIGURE 4:**
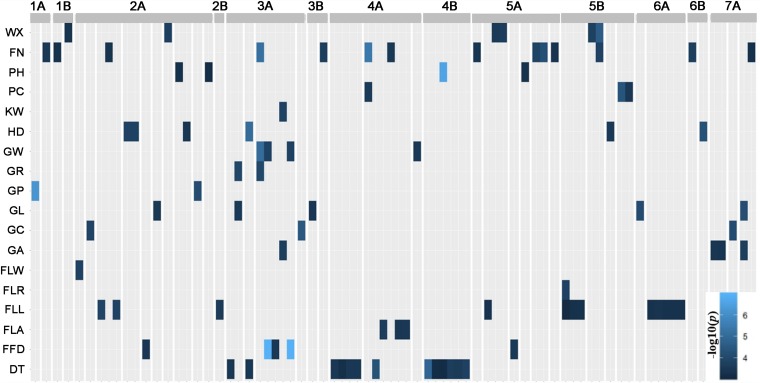
Marker-trait associations (MTAs). The GWAS detected 90 MTAs associated with 17 of the 18 morphological traits and DT. Based on a threshold marker LD (*r*^2^) > 0.2, the MTAs represent 44 distinct genomic regions.

**FIGURE 5:**
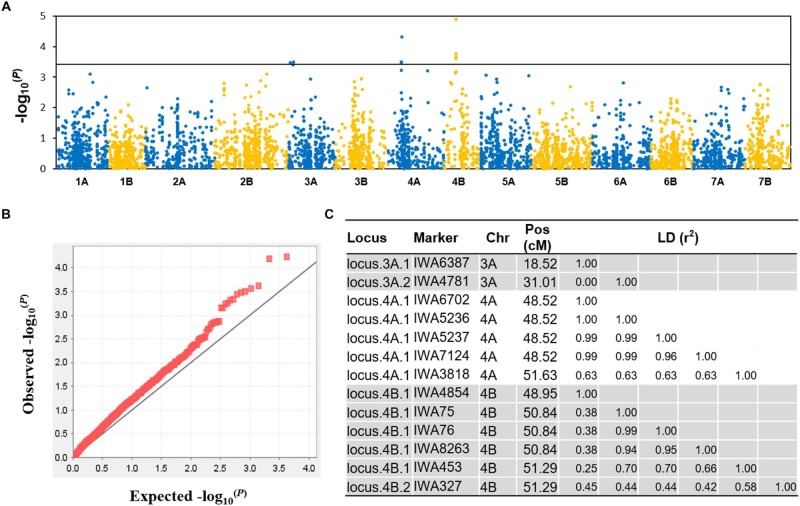
GWAS for DT. **(A)** A Manhattan plot showing the location and –log10(p) value of the markers, **(B)** a quantile-quantile plot illustrating the coincidence between observed and expected –log_10_^(P)^ values expected for the tail, indicating an appropriate population stratification, **(C)** haplotype blocks or single markers significantly associated with DT.

The GWAS results are summarized in Supplementary Table S3. The quantile-quantile (QQ) plots (Supplementary Figures S3, S4) revealed that most of traits fitted the model well, although FLW was over-corrected. The genomic distribution of marker-trait-associations is shown in Figure 4. For DT, as an example, the Manhattan plot, quantile-quantile plot and haplotype blocks are given in Figure 5. In all, the GWAS identified 90 marker-trait-associations (MTAs) covering all but one of the traits (the exception was PL). The proportion of the phenotypic variation explained (PVE) by each MTA varied from ∼2–6%. The highest number of MTAs per trait (14) was associated with SN, followed by 13 for DT and 12 for FLL; each of the remaining traits was associated with fewer than 10 MTAs. The set of MTAs was defined by 78 markers, of which 67 accounted for a single trait, 10 for two traits and one for three traits. The 78 markers could be further merged into 44 loci according to their chromosome location and LD (*r*^2^ > 0.2), 10 of which were relevant to more than one trait. The number of loci detected and the total PVE highly varied, where more loci were associated with SN (12), HD (6), DT (5), FLL (5), GL (5) and total PVE from 16 to 39%; and other traits were less associated with less than five loci and total PVE from 3 to 14%.

A set of markers used to define nine regions accounted for variation in more than one of the various grain morphology traits, while the co-location of MTAs associated with two or more unrelated traits also occurred (Supplementary Table S3). For example, the region denoted locus.4A.2 harbored MTAs relating to DT, FLA, and SN. The other example is the locus.4B.1 harboring MTAs related to both DT and PH. With respect to plant stature, this may arise as a result of the nearby semi-dwarfing gene *Rht-B1* (*TraesCS4B01G043100*, 30,861,382 to 30,863,247 bp) in the locus.4B.1 region (Supplementary Table S4). The alleles inherited from the more drought tolerant entries at the six markers which defined the locus.4B.1 region were all associated with a reduction in PH, although only *IWA4854* exceeded the chosen significance threshold (*P* < 0.001).

### QTL or Candidate Genes Underlying GWAS Loci

Based on sequence alignment of markers to the reference pseudomolecules (International Wheat Genome Sequencing Consortium, 2018), the detected loci by GWAS were compared with previously reported QTL and/or major genes (Supplementary Table S4). The consensus loci included those on chromosomes 4B and 5A for PH, 2A and 5B for HD, 1A for GP, 5B for WX, 4A for FLA, 5B for PC, 3A and 6A for grain morphology traits, 3A and 4B for DT. However, only two of the underlying genes were verified in wheat, including *Rht-1B* for locus.4B.1 (PH) and *Ppd-A1* for locus.2A.5 (HD). In addition, three MTAs, include IWA2023 on 3A for SN, GW, and GR, IWA2816 on 4A for SN, and IWA4363 on 7A for SN, GL, and GA, were validated through linkage mapping in two F_2:3_ families with the parents selected from the association panel (Supplementary Figure S5). Results from the QTL analysis were highly comparable with that from the GWAS, indicating the GWAS results were quite reliable.

Candidate genes were analyzed through gene annotation combined with their homology to functionally characterized genes in model plants or other cereals, and also their expression pattern (Supplementary Table S4). A total of 14 putative candidate genes involved in enzymes, hormone-response, and transcription factors were identified. Some of them have been verified in wheat and other related phenotypes in model plants or other cereals.

**FIGURE 6:**
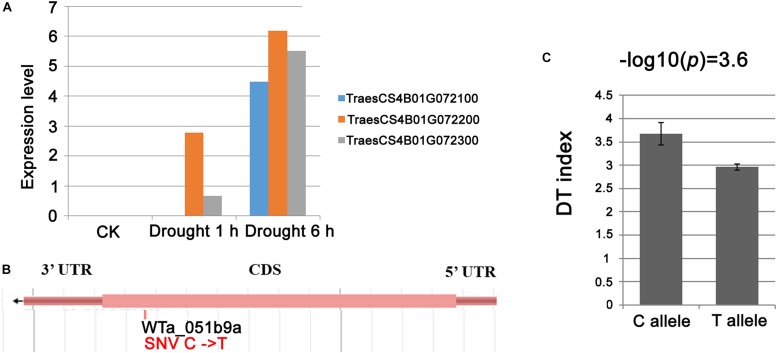
The *WD40* genes were candidates for drought tolerance of locus.4B.1. **(A)** Drought induced expression of the three *WD40* genes *TraesCS4B01G072100*, *TraesCS4B01G072200*, and *TraesCS4B01G072300* underlying locus.4B.1; **(B)** the site of SNP (WTa_051b9a) at the tale of *TraesCS4B01G072200* lead to premature stop of protein translation; **(C)** significant association between SNP WTa_051b9a and DT index in durum panel.

The genetic region for locus.4B.1 was also reported to be associated with drought tolerance by Kadam et al. (2012), and this region corresponded to an ∼16 Mb genomic interval harboring a set of an estimated 117 genes (Supplementary Table S5). Of the 117 genes present in this segment, only six were transcriptionally affected by the drought treatment: *TraesCS4B01G071500* (predicted to encode an α subunit of pyruvate dehydrogenase E1), three tandemly arranged genes *TraesCS4B01G072100*, *TraesCS4B01G072200*, and *TraesCS4B01G072300* (WD40 family protein), *TraesCS4B01G076400* (Adenine/guanine permease), and *TraesCS4B01G077900* (gibberellin-regulated protein 1). Among the six genes, the 4D homeolog of the three WD40 protein coding genes was shown by Kong et al. (2015) to confer tolerance to exogenously provided abscisic acid, salinity stress and osmotic stress, suggesting that the *WD40* genes might be candidates for DT of locus.4B.1. Sequence analysis of the three WD40-coding genes indicated a C-T SNP present in the coding sequence of *TraesCS4B01G072200*, which lead to premature stop of translation. In addition, a mixed linear model was applied on this variation by taking the top markers from other DT-related loci, population structure and kinship in consideration, the result showed that the premature stop variation was significantly associated with the drought sensitive phenotype in the durum panel (Figure 6).

### DT-Related Haplotypes of Locus.4B.1 in Durum and Bread Wheat

**FIGURE 7:**
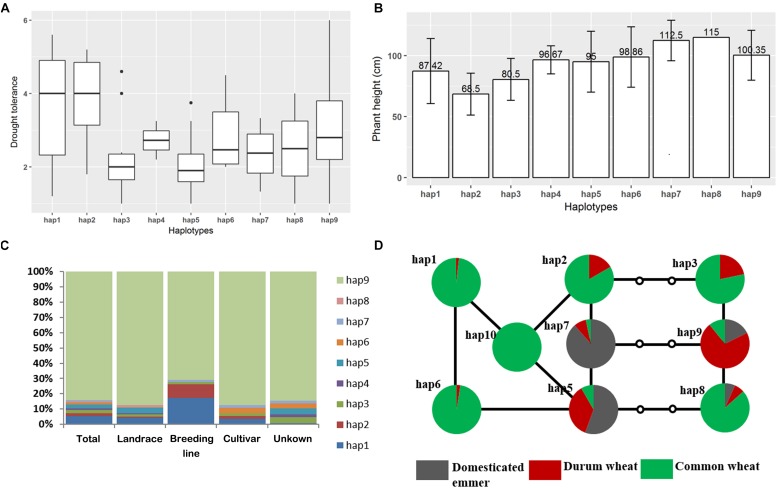
Haplotype analysis in the 4B.1 region. **(A)** Variation in the trait between the nine haplotypes revealed in the durum wheat panel, **(B)** the influence of haplotype on plant height, **(C)** haplotypes represented in emmer wheat, durum wheat and bread wheat, **(D)** a haplotype network showing the nine major haplotypes present in emmer wheat, durum wheat and bread wheat. Empty circles indicate sequences not uncovered in the current materials.

As locus.4B.1 region was previously identified as a major-effect DT-related QTL in bread wheat and also detected in this study, we suggest it is an important DT-related QTL in durum wheat. To gain a further insight into the genetic variation of this region and their relationship with DT, we conducted haplotype analysis to this region. The results showed a total of 9 haplotypes (hap1–9) in durum wheat, with hap9 carried by 84% of entries in the durum wheat panel (Supplementary Tables S6, S7). With respect to the DT index, two minor haplotypes, hap1 and hap2, were significantly tolerant for DT than the dominant haplotype hap9 and the other 6 minor haplotypes (Figures 7A–C). The frequency of hap1 and hap2 was noticeably higher in the improved materials (and particularly in the breeding lines) than in the landraces, implying a positive selection for these two haplotypes carried out by durum wheat breeders (Figure 7C).

A similar analysis was carried out in the emmer wheat and the bread wheat panel, respectively. In the emmer wheat panel, the most common haplotypes were hap9, hap7 and hap5, with hap8 present in just two entries. The bread wheat panel was dominated by hap1 (72%) and hap6 (15%), and added a further six minor haplotypes (hap10 through hap15) (Supplementary Tables S6, S7). A haplotype network established for the nine major haplotypes in the three diversity panels revealed a separation between two sets of haplotypes: the first grouped hap1, hap2, hap5, hap 6, hap7, and hap10, and the second hap3, hap8, and hap9 (Figure 7D). Two haplotypes of the locus.4B.1 region dominated the set of emmer wheats, with hap9 represented in entries of the southern sub-population while hap7 was carried by numerous entries of the northern sub-population of emmer wheat. As the dominant haplotypes hap1 and hap6 in bread wheat was more closely related to hap7, while durum wheat was dominated by hap9, we can predict that the durum wheats were more closely related to the southern sub-population emmer wheats while modern bread wheats were more closely related to the northern ones.

## Discussion

### The Genetic Basis of Agronomic Traits and DT in Durum Wheat

In this study, we identified 44 chromosome loci associated with 17 agronomic traits and a drought wilting score using GWAS on a durum wheat panel. For a given trait, the total PVEs varied from 16 to 39%, illustrating that most of the traits were under polygenic control. Similarly, previous GWAS and QTL analysis has shown that most of the traits investigated here, including PH, HD, WX, flag leaf-, spike- and grain-associated traits are controlled by multiple genes, each contributing only modestly to the overall genotypic variance (Gegas et al., 2010; Zhai et al., 2016; Liu et al., 2018). Surprisingly, both GP and GC fell into this category, even though both traits are relatively simply inherited (Khlestkina et al., 2006). A possible explanation for this unexpected result, which was also observed in a similar analysis of the genetic basis of awn type in bread wheat (Liu Y. et al., 2017), is that GWAS imposes genetic stratification on the population, which masks the effect of major genes. Correlation between agronomic traits was widely observed in previous studies, which can be partially explained by the presence of pleiotropic genes (e.g., Liu Y. et al., 2017; Roncallo et al., 2017; Schulthess et al., 2017). Twelve of the 78 SNP markers (15%) and 10 of the 44 genomic loci (23%) were relevant to more than one trait, which confirms the frequent observation that genes responsible for variation of significant agronomic traits express extensive pleiotropy and/or linkage. Some recent examples have been documented in bread wheat and durum wheat (e.g., Liu Y. et al., 2017; Roncallo et al., 2017; Schulthess et al., 2017).

A number of the genomic sites associated with a trait coincided with the location of previously discovered QTL and/or major genes (Supplementary Table S4). The sites of two of the three loci associated with PH, for example, were consistent with that of the height reducing genes *Rht-B1* (Peng et al., 1999) and *Rht9* (Ellis et al., 2005). We found that the diversity of *Rht9* in durum wheat was higher than that of *Rht-B1*, the dwarfing gene employed in bread wheat during the Green Revolution, suggesting that *Rht9* might play more important roles than *Rht-B1* in durum genetic improvement. HD or flowering time in wheat is dependent on the array of genes controlling a cultivar’s requirement for vernalization (*Vrn* genes) and photoperiod (*Ppd* genes), in addition to a number of genes promoting earliness *per se* (*Eps* genes). A GWAS study has identified the dependence of bread wheat’s flowering time on the identity of the *Ppd-D1* allele present and on the gene copy number at *Ppd-B1*, with a number of minor effect loci also contributing (Würschum et al., 2018). We observed a positive GWAS signal coinciding with the location of *Ppd-A1* rather than that of *Ppd-B1*, consistent with observations made by Maccaferri et al. (2010). There was no such signal coincident with any of the *Vrn* loci, presumably because none of the entries have any vernalization requirement. In all, durum wheat possessed more loci for controlling flowering time than bread wheat, and the combination of different loci enhanced the diversity of flowering time in durum, which is possibly helpful for adapting to the diverse environment of Mediterranean region. The MTAs mapped to sites that were consistent with the literature and included those on chromosomes 1A (GP), 5B (WX), 4A (FLA), 5B (PC), 3A and 6A (grain morphology traits), and 3A and 4B (DT). The discovery of a large number of non-coincident MTAs may simply reflect the extent of the divergence between durum wheat and bread wheat, since the bulk of the genetic analyses reported in the literature relate to bread, rather than to durum wheat.

### Gene Candidature Under the GWAS Loci

The level of gene annotation currently available has allowed some tentative predictions to be made of the identity of the genes underlying variation in some traits (Supplementary Table S4). *TraesCS2A01G540400*, a putative *GA3ox2* (gibberellin 3-oxidase 2) gene, was a primary candidate for the PH-related locus.2A.13. Its close homologs in maize (*ZmGA3ox2/qPH3.1*) and rice (*OsGA3ox2/d18*) have been verified to function for PH (Itoh et al., 2001; Teng et al., 2013). Potential candidates for the HD MTAs on chromosomes 2A are *TraesCS2A01G269700* (*flowering promoting factor-like 1*). Previous reports in Arabidopsis and rice have highlighted the critical role of cytokinin oxidase (CKXs) to promote flowering or seed number (Werner et al., 2006). Potential candidates for the chromosomes 1B, 3B, and 7A SN MTAs are *TraesCS1B01G176000*, *TraesCS3B01G344600*, and *TraesCS7A01G536900*, respectively, all of which have been assigned as coding genes for CKXs. Finally, as aforementioned, the location of the DT MTA on chromosome 4B coincided with that of the tandem array of WD40-encoding genes. The present evidence indicated that genes homologous to that functionally characterized in model species or other cereal crops showed candidatures for some of the GWAS loci, suggesting they are good targets for more detailed analysis.

### Rare Variants of Locus.4B.1 Containing Candidate Genes Enhanced DT in Durum Wheat

Drought represents a major constraint over the yield of bread wheat (Fleury et al., 2010), and is of particular importance to durum wheat given that its prime areas of production are located in regions of low rainfall (Habash et al., 2009). Previous studies revealed that the region around the semi-dwarf gene *Rht-B1* on chromosome arm 4BS associated with multiple traits responsible for tolerance to drought stress in bread wheat (Kadam et al., 2012). The QTL with major DT-related effect was also found in the collinear chromosome regions in barley on chromosome 6H, rice on chromosome 1 and maize on chromosome 3 (Swamy et al., 2011). In this study, we confirmed the presence of a DT-related QTL (locus.4B.1) in this region downstream of *Rht-B1* in durum wheat by GWAS.

Among the existing 9 haplotypes of locus.4B.1, only two rare ones (hap1 and hap2) are related to the DT phenotype in durum wheat (Figure 7A). Both DT haplotypes show potential for application in breeding DT durum cultivars as they are possessed by only a limited number of entries (Figure 7C). In rice, breeding selection on *SD1* significantly reduced *qDTY.1.1* due to linkage drag (Vikram et al., 2015). However, there’s no evidence that favorable alleles from *Rht-1B* and locus.4B.1 were linked in a repulsion fashion in durum. In contrast, DT alleles from all the six markers defining the locus.4B.1 also associated with reduced PH. This indicates a large proportion of the entries may possess dwarf-alleles and DT-alleles from each locus. The linkage between DT and reduced stature may provide an advantage for promoting these two traits together in breeding.

Three tandemly repeated *WD40* genes under locus.4B.1 are suggested as primary candidate genes for DT according to three clues. First, each of the genes was significantly up-regulated by exposure to drought; second, their 4D homeolog is known to confer tolerance to osmotic stress (Kong et al., 2015); and third, the members of the *WD40* family have been identified as functioning in the plant stress response (Sharma and Pandey, 2016; Liu W.C. et al., 2017). In this work, a SNP resulting in a translational truncation in the second 4B *WD40* (*TraesCS4B01G072200*) gene was found to be significantly associated with reduced DT (Figure 6). Furthermore, the drought-induced expression of *TaWD40s* was much depressed when combined with heat stress (Supplementary Table S5), revealing a potential relatedness to the enhanced stress effect when heat and drought stresses are integrated. Therefore, all above evidences indicate that the *WD40* genes are associated with DT QTL in wheat and are worth further analysis.

## Conclusion

In this manuscript, we presented an insight into the worldwide genotypic and phenotypic diversity in durum wheat, as well as the genetic control for major agronomic traits and drought resistance. The highly phenotypic variation and correlation were shown to be controlled by a large number of genomic loci with multi-polygenic and pleiotropic effect. A few candidate genes were selected by comparable genomic analysis plus their expression pattern and genomic variation. In addition, the haplotype investigation in a DT-related loci locus.4B.1 showed potential application in breeding DT durum cultivars, as well as a complex evolutionary history and gene candidature. All these data are relevant in the context of durum wheat improvement and the isolation of genes underlying variation in some important quantitative traits.

## Data Availability

All datasets for this study are included in the manuscript and the Supplementary Files.

## Author Contributions

This study was designed by GX, SL, and SW. The evaluation of traits was conducted by SW, SL, and SX. The emmer wheat panel was collected by SX. The genotypic evaluation of the durum wheat and emmer wheat panels was carried out by SC and QS. Data were analyzed by SW. The manuscript was drafted by SW and SL, and revised by GX. All authors have reviewed and approved the final version of the manuscript.

## Conflict of Interest Statement

The authors declare that the research was conducted in the absence of any commercial or financial relationships that could be construed as a potential conflict of interest.

## Footnotes

1 https://www.ndwheat.com/buyers/NorthDakotaWheatClasses/Durum/

2 https://triticeaetoolbox.org/wheat/

3 https://www.usclimatedata.com/climate/casselton/north-dakota/united-states/usnd006

4 https://imagej.nih.gov/ij/

5 https://cran.r-project.org/package=genetics

6 https://wheat-urgi.versailles.inra.fr/Seq-Repository/Assemblies

7 https://web.stanford.edu/group/pritchardlab/structure.html

8 https://www.broadinstitute.org/haploview/haploview

9 https://cran.r-project.org/package=qtl

## Supplementary Material

FIGURE S1 Inter-trait correlations among 8 drought related traits tested on 15 durum wheat lines. Pearson’s correlation coefficients are shown in the lower panel and significant correlations (*p* < 0.01) are labeled with gray. Experimental repeatability is shown in parentheses after each trait in the diagonal. DT, drought wilting score; CC, chlorophyll content; RI, leaf rolling index; WC, leaf water content; SR, seedling survival rate; DSI, drought susceptibility index; TN, tiller number; SN, seed number per spike; GW, thousand grain weight. CC, RI, and DSI were measured according to Peleg et al. (2009). WC was estimated according to Kong et al. (2015).

FIGURE S2 Frequency distribution for the 18 morphological traits and DT among the members of the durum wheat diversity panel.

FIGURE S3 Qantile-quantile plots for the GWAS results from 9 agronomic traits.

FIGURE S4 Qantile-quantile plots for the GWAS results from the other 9 agronomic traits.

FIGURE S5 Verification of three MTAs through linkage mapping. QTL analysis was conducted in the F_2_ population PI520392/PI210912 targeting MTAs IWA2023 on 3A for SN, GW, and GR, IWA2816 on 4A for SN **(a,b)** and in the F_2_ population PI191571/CITR14814 targeting MTA IWA4363 on 7A for SN, GL, and GA **(c)**. Markers corresponding to the MTA detected by GWAS were colored with red. PVE, phenotype variation explained by the peak marker.

TABLE S1 Markers used in linkage analysis.

TABLE S2 The summary of provenance, breeding status and population structure inferred by the admixture model in the STRUCTURE software package of the durum wheat diversity panel.

TABLE S3 Markers significantly associated with traits identified by GWAS.

TABLE S4 Reported QTL and candidate genes underlying GWAS loci.

TABLE S5 The presumed function of candidate genes for the DT trait located in the 4B.1 region.

TABLE S6 Haplotypes present in the 4B.1 region among the durum wheat, emmer wheat and bread wheat materials.

TABLE S7 The distribution of haplotypes presents in the 4B.1 region among the durum wheat, emmer wheat and bread wheat materials.
