# Supplementary figures and images for: A Genome-Wide Association Study of Highly Heritable Agronomic Traits in Durum Wheat
Source: Front Plant Sci. 2019 Jul 17;10:919. doi: 10.3389/fpls.2019.00919 (PMC6652809; doi:10.3389/fpls.2019.00919)

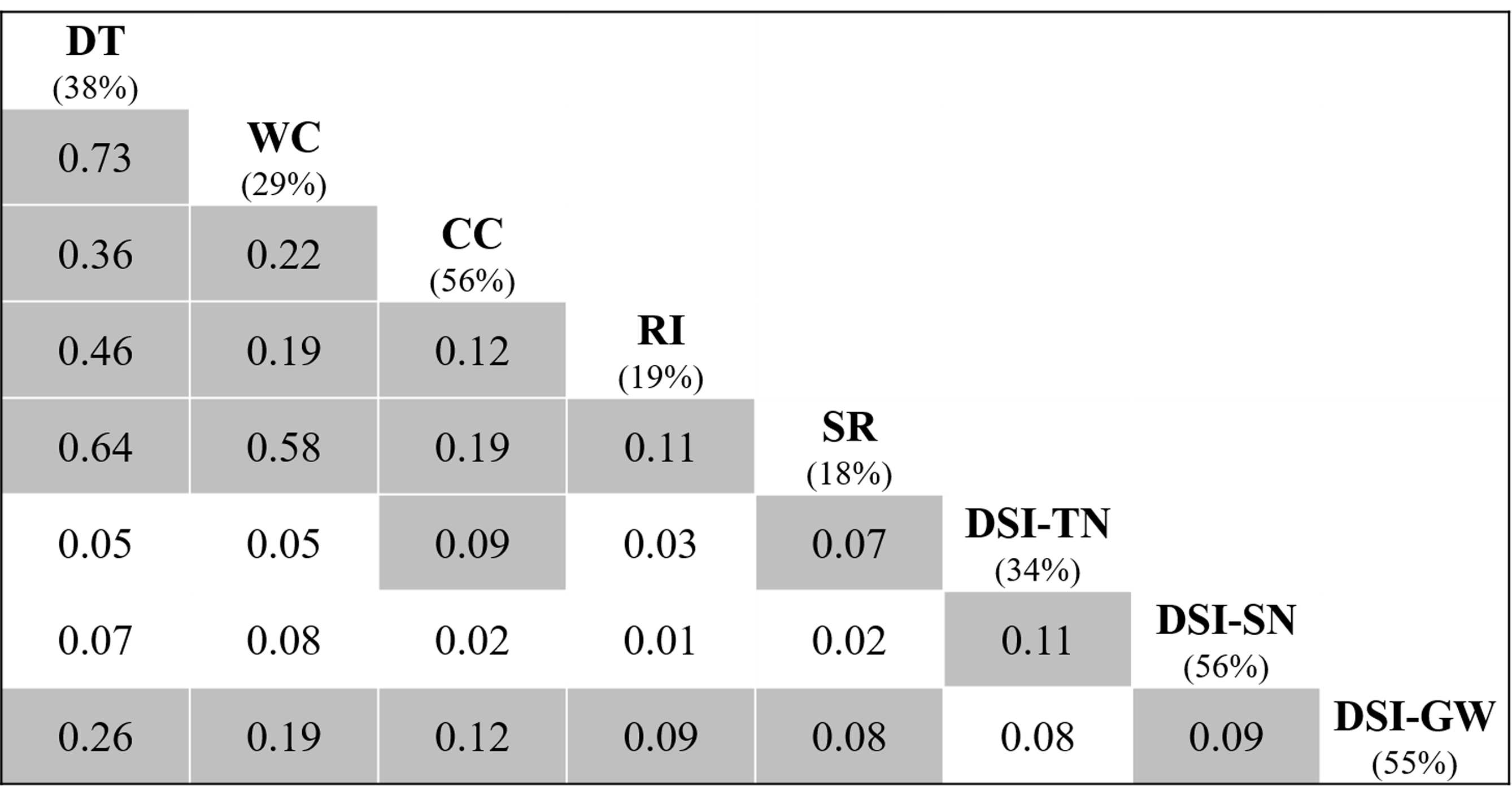

Supplement: FIGURE S1 — Inter-trait correlations among 8 drought related traits tested on 15 durum wheat lines. Pearson’s correlation coefficients are shown in the lower panel and significant correlations (p < 0.01) are labeled with gray. Experimental repeatability is shown in parentheses after each trait in the diagonal. DT, drought wilting score; CC, chlorophyll content; RI, leaf rolling index; WC, leaf water content; SR, seedling survival rate; DSI, drought susceptibility index; TN, tiller number; SN, seed number per spike; GW, thousand grain weight. CC, RI, and DSI were measured according to Peleg et al. (2009). WC was estimated according to Kong et al. (2015). [file Image_1.TIF]

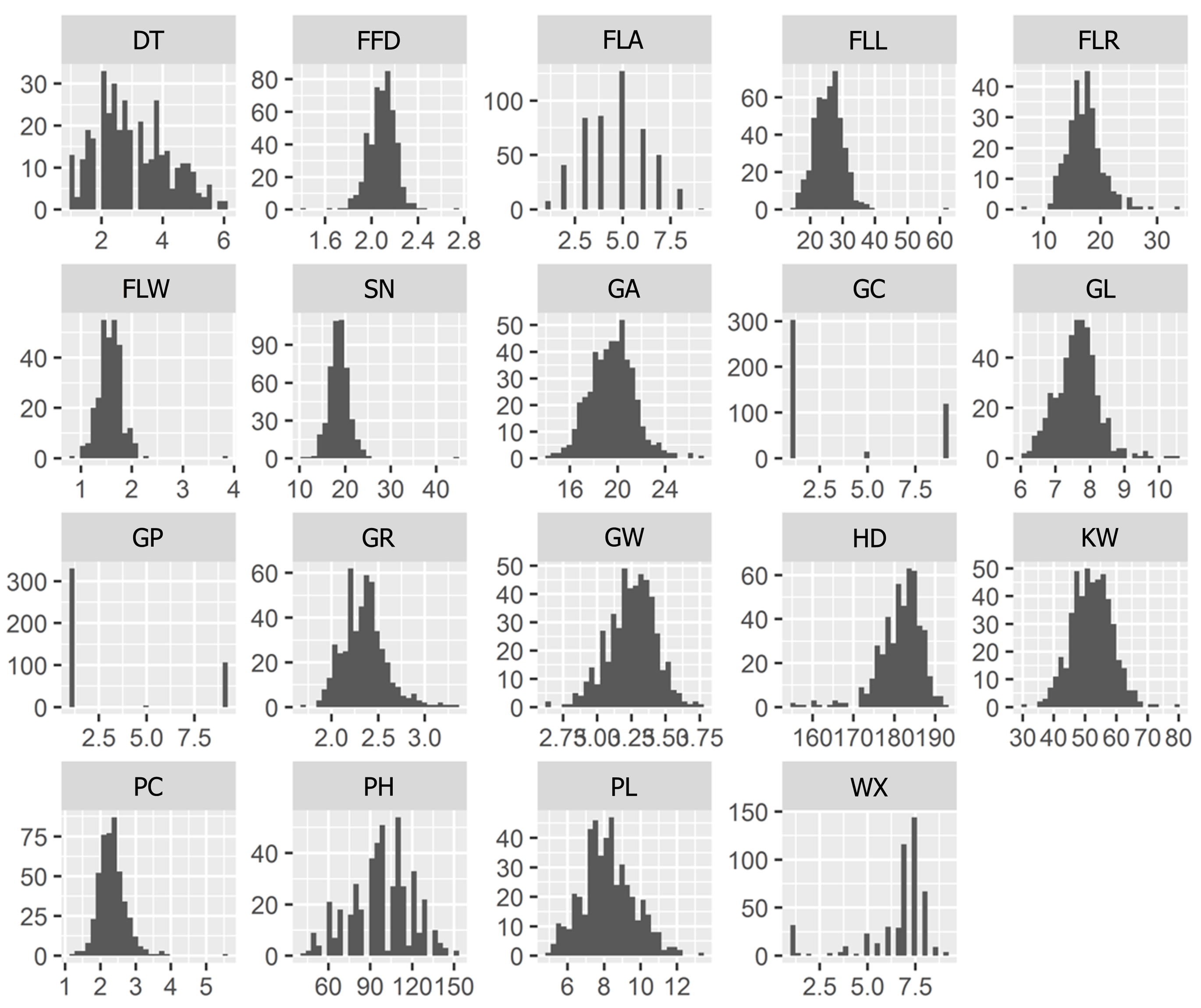

Supplement: FIGURE S2 — Frequency distribution for the 18 morphological traits and DT among the members of the durum wheat diversity panel. [file Image_2.TIF]

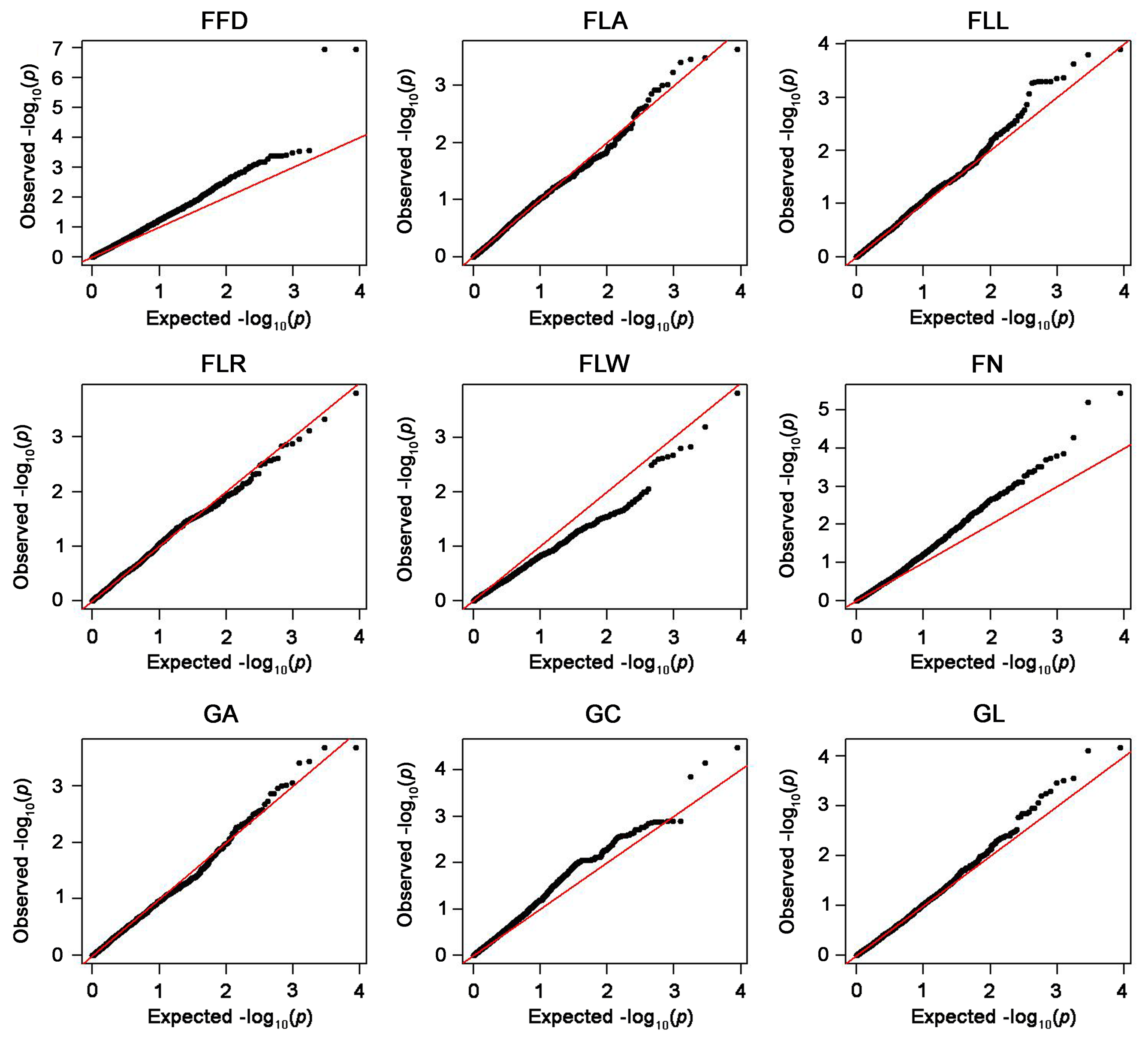

Supplement: FIGURE S3 — Qantile-quantile plots for the GWAS results from 9 agronomic traits. [file Image_3.TIF]

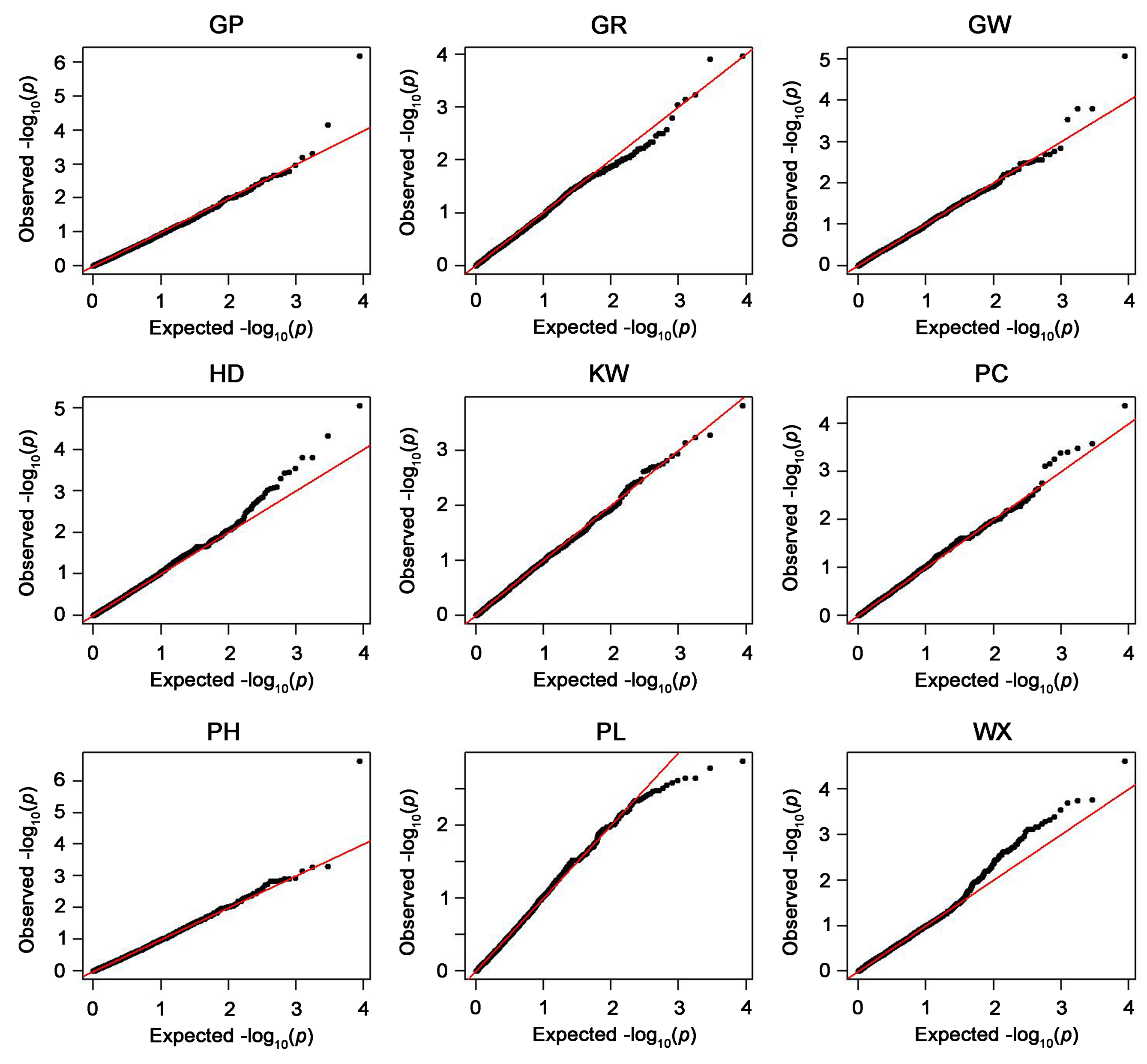

Supplement: FIGURE S4 — Qantile-quantile plots for the GWAS results from the other 9 agronomic traits. [file Image_4.TIF]

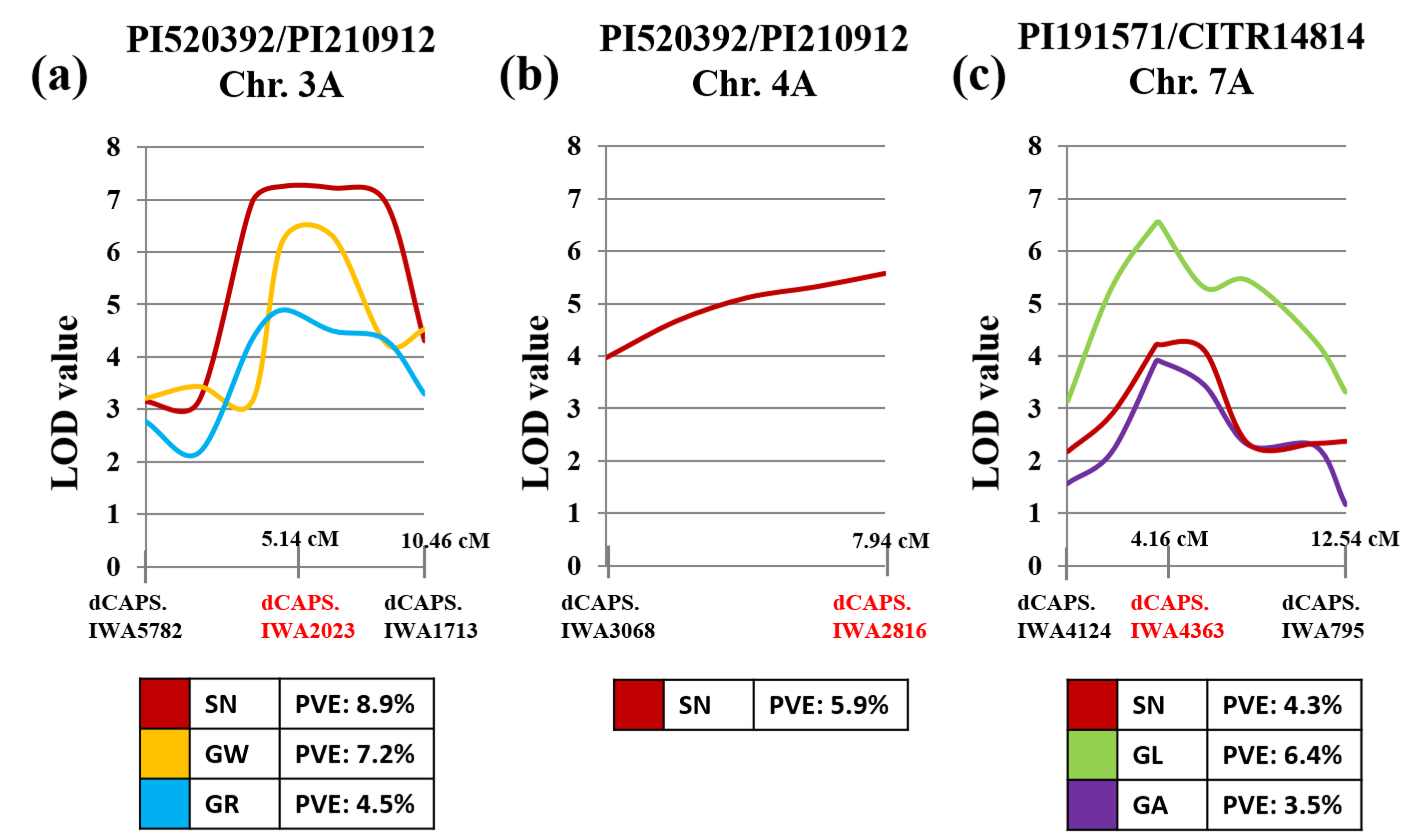

Supplement: FIGURE S5 — Verification of three MTAs through linkage mapping. QTL analysis was conducted in the F2 population PI520392/PI210912 targeting MTAs IWA2023 on 3A for SN, GW, and GR, IWA2816 on 4A for SN (a,b) and in the F2 population PI191571/CITR14814 targeting MTA IWA4363 on 7A for SN, GL, and GA (c). Markers corresponding to the MTA detected by GWAS were colored with red. PVE, phenotype variation explained by the peak marker. [file Image_5.TIF]
